# Parental support and bullying bystander behaviors in Chinese adolescents: Longitudinal mediation through social harmony

**DOI:** 10.3389/fpubh.2023.994658

**Published:** 2023-03-10

**Authors:** Xiaoyu Jia, Jun Wang, Yuchi Zhang

**Affiliations:** ^1^College of Teacher Education, Southwest University, Chongqing, China; ^2^Key Laboratory of Child Cognition & Behavior Development of Hainan Province, Haikou, China; ^3^Chinese Department, Qiongtai Normal University, Haikou, Hainan, China; ^4^Department of Educational Technology, School of Smart Education, Jiangsu Normal University, Xuzhou, China

**Keywords:** bullying bystander, bullying, parental support, social harmony, cultural value, Chinese culture

## Abstract

**Objectives:**

Bullying in schools is a serious concern worldwide. The active defending or passive bystanding behaviors of bullying bystanders significantly contributes to the prevention of bullying. Relevant studies have increasingly adopted a social-ecological system approach in bullying research. However, the role of parental factors (microsystem) and cultural value (macrosystem) factors in adolescents' bullying behaviors in non-western culture contexts is unclear. Social harmony, which is closely related to social behavior, is a core value in Chinese culture. Exploring the role of social harmony in bullying bystanders in China could enhance our understanding of bullying, and enrich the diversity of the literature. This study aimed to examine the mediation effects of social harmony on the associations between parental support and the bullying bystanders among Chinese adolescents.

**Materials and methods:**

The participants comprised 445 Chinese adolescents (mean age = 14.41, *SD* = 0.51) from Beijing City, China. A 17-month, two-point longitudinal study was conducted. Parental support, social harmony, and the behavior of bullying bystanders were evaluated at two time points. The hypothesized mediation model was examined using a structural equation modeling approach using bootstrapping techniques.

**Results:**

The results showed that social harmony partly mediated the positive relationship between adolescents' parental support and active defending behaviors, and fully mediated the negative relationship between adolescents' parental support and passive bystanding behaviors.

**Conclusion:**

These results highlight the importance of studying parental and cultural values in research on bullying bystanders.

## 1. Introduction

Bullying, a subcategory of aggression characterized by “intentionality, repetition, and an imbalance of power” (1), has increased in schools worldwide, resulting in widespread concern in societies (1, 2). Bullying is significantly associated with adolescents' poor physical health (e.g., sleep quality) and mental health (depression, anxiety, and suicide attempts) outcomes in the short- and long-term (2–6). A five-decade longitudinal study found that being a victim of bullying during childhood was associated with poorer general health in adulthood (7). Being bullied in adolescence was positively related to increased internalization of problems in adulthood (8). Moreover, bullying presents a public mental health problem, resulting in a large financial cost burden on the government (5, 9, 10). For example, in England, bullying and harassment are estimated to cost taxpayers approximately $2.281 billion annually (9). Following previous studies (11, 12), this study distinguishes between two different kinds of bystander behavior when witnessing bullying at school: active defending behaviors, which are behaviors that may stop bullying or protect victims from bullies (e.g., intervening to stop the bullying, asking for an adult or teacher's help), and passive bystanding behaviors, which are behaviors that “withdraw from the scene, deny any bullying is going on, or remain as a silent audience” to bullying (11). Numerous studies have shown that the behavior of bystanders during bullying can either reduce bullies' harm (active defending behaviors in this study, such as stopping bullies or calling adults for help) or aggravate harm (passive bystanding behaviors, such as minding their own business or avoiding bullying incidents) (11, 13, 14). Many intervention programs have contributed to effectively reducing the prevalence of bullying through systematic training for bullying bystanders, highlighting their vital role in preventing bullying (15, 16). In a meta-analysis, bullying bystander interventions were shown to effectively reduce bullying prevalence in schools (17). Thus, exploring the antecedent variables and potential mechanisms of bullying bystander behavior has important global theoretical and practical implications. Parents are considered the most important factor in adolescents' development and are closely related to their socialization processes and social behavioral outcomes (18–21). As the primary source of social support for adolescents' social lives, parental social support is related to numerous social development outcomes (20, 22) in adolescents. Therefore, this study focused on its role in adolescents' bullying bystander behaviors in school contexts. Recently, the role of parental factors in the behavior of different bullying bystanders has been highlighted in a few studies (23–26). Related research has found that parental support and attachment security are closely related to active and passive bullying bystander behaviors (25).

However, there are several gaps in the literature. First, only a few studies have explored the association between parental social support and bullying bystander behaviors, and none have used the social ecological system theory approach to fully explore the potential mechanisms (18). Despite researchers arguing nearly two-decades ago that bullying issues should be viewed from a social-ecological perspective that considers the combined effects of family, cultural values, and other factors, the complex effects of parental and cultural values in bullying bystanders still lack full recognition today (27). According to the social ecological system theory, adolescents' developmental outcomes are the result of complex and combined effects of different ecological system factors (28). Parental-related factors (i.e., parental social support in the present study) are the most proximal and profound microsystem factors in adolescents' development, which may have either a direct or indirect influence on their social behaviors (28). Moreover, social harmony, a macrosystem-level factor (cultural value or social norms) that refers to “the extent to which an individual values peaceful and concordant interpersonal relationships (or social stability),” is one of the most significant and deeply influential Confucian values in Chinese society (29). Merging the value acquisition model and Schwartz's values theory, high-quality social support from parents could facilitate the development of children's socialization and internalize social mainstream cultural values (social harmony) (30–32). A higher endorsement of social harmony may lead to more active defending behaviors and less passive bystanding behaviors.

Second, most empirical studies focus on active defending behaviors but overlook passive bystanding behaviors and their antecedent variables (33–35). However, peers' passive bystanding behavior can aggravate bullying processes (11). It is important to explore the association between parental support, active defending behaviors, and passive bystanding behaviors in bullying.

Third, most bullying studies were conducted in Western countries (14). Although several studies have explored bullying in collectivistic cultural contexts, none have closely examined the potential mediating role of social harmony in the Chinese cultural context (36, 37). Social harmony is the core value of Chinese Confucianism, which has a profound influence on Chinese social lives. Schwartz's values theory and previous studies on collectivist societies have not demonstrated the role of social harmony, which has a distinct origin in the collectivism (38). It is important to examine Schwartz's value theory in China (30, 39). Exploring the potential mediating role of social harmony in the Chinese cultural context could contribute to enriching the theoretical framework of bullying from culturally diverse groups, and could also provide evidence for school or local authorities to develop more culturally sensitive and tailored bullying bystander interventions.

Fourth, most studies have adopted a cross-sectional design, failing to examine causal relationships, while a limited number of studies have collected only short-term longitudinal data.

Based on social ecological system theory, the value acquisition model, and Schwartz's values theory, this study aims to fill these gaps in the research by examining the mediating role of social harmony value in the links between parental support and the active defending and passive bystanding behaviors of Chinese adolescents through an 17-month longitudinal study.

### 1.1. Parental support and bullying bystander behaviors in school

Parents are important social agents who play a critical role in adolescents' socialization and social behavior development (38, 40). Perceived parental support (due to the social background in mainland China, all parents in our study are heterosexual couples) in the present study can be defined as adolescents' perceived level of being “cared for, loved, esteemed, and valued… [as] a member of a network of common and mutual obligation” from their parents (41). Perceived parental support is a major source of social support in adolescents' lives (42). Based on attachment theory, warmth and responsive social support from parents may facilitate the secure attachment and internal working models of children (43). Consequently, adolescents may develop more prosocial behaviors in interpersonal processes (44–46). For example, a cross-sectional study of 306 US Latina/o adolescents revealed that higher parental support is positively related to prosocial behavior among adolescents (44). However, only one cross-sectional empirical study has examined the relationship between parental support and active defending behaviors in bullying (25). Active defending behaviors are a form of prosocial behavior (47); therefore, it is natural to assume that warm parental support may foster more active defending behaviors among bullying bystanders.

On the other hand, according to attachment theory, a lack of parental support may hinder the facilitation of secure attachment (44, 45). Adolescents who perceived less or negative parental support may hardly trust or seek advice from their parents. This type of internal working model may guide adolescents to avoid engaging in bullying incidents to protect themselves (43). Moreover, low parental support is associated with lower self-efficacy, more serious emotional regulation difficulties, and more biased social information processing among adolescents that may relate to antisocial or delinquency behaviors (48). Adolescents with low parental support who witness bullying incidents may be more likely to pretend not to see them when they occur.

To our knowledge, no empirical study has explored the relationship between parental support and adolescents' active defending and passive bystanding behaviors simultaneously. This study aimed to explore these associations to enhance our understanding of this phenomenon.

### 1.2. Mediating role of social harmony

The behavior of bullying bystanders is facilitated by multiple ecological system-level factors that encompass direct family factors (microsystem factors) and cultural values or beliefs (macrosystem factors) (14). Therefore, adolescents' bullying bystander issues may not be fully understood without exploring both the family and cultural contexts involved in the phenomenon (27, 28, 49, 50). Adolescence is a key phase in the formation of cultural values or beliefs (51). Parents significantly contribute to the development of cultural values among adolescents.

The Chinese are deeply influenced by Confucian philosophy and cultural values (29, 38). Social harmony is recognized as a prominent cultural value that facilitates positive interpersonal relationships (22, 52). Confucianism emphasizes that individuals should try to maintain their relationships with others peacefully and reduce interpersonal conflict. Individuals should try to avoid behaviors that threaten social harmony, and prosocial behaviors to prevent the disruption of social harmony by others are also recommended (38). If one could help groups to maintain social harmony then he or she will be considered a “Junzi” (a man of noble character), one of the highest recognized marks of great moral character in traditional Chinese culture (53). In addition, although the social harmony value coincides with the concept of a collectivist culture, “social harmony has a distinct origin from collectivism in Chinese culture,” deeply rooted in Chinese Confucian philosophy (53, 54). Hence, social harmony may not play as critical a role in other collectivist societies (38). Moreover, most measures of collectivism do not include distinct items evaluating social harmony (13, 55). Thus, it is urgent to explore the potential role of social harmony among Chinese adolescents in social behaviors, which will further extend the literature on bullying and improve cultural sensitivity in related research fields.

Based on the value acquisition model, warm parental social support is the core mechanism through which children successfully internalize mainstream cultural values (31). Parental social support may lead to greater endorsement of social harmony among Chinese adolescents. However, no empirical study has examined these associations. Previous studies partly support this assumption, finding that parental support positively influences the transmission of cultural values, as parental warmth and supportive behaviors with high cohesion and low conflict may facilitate children's positive perception of their parental message and acceptance of cultural values (31, 32). For example, the quality of positive parental practices is related to higher collectivist and individualist values among adolescents (20). Therefore, parental social support may be positively related to higher social harmony.

The guidance function of cultural values for individual behavior is well-established (30, 38). According to Schwartz's values theory, an individual's value-consistent action is rewarding (30). Consequently, children who endorse higher social harmony values may exhibit more prosocial behaviors that could promote social harmony. Active defending behaviors may help victims stop the bullying process that threatens harmony in peer groups (29, 52, 56). Thus, it can be assumed that social harmony is positively related to more active defending behaviors in bullying. On the other hand, research has also found that lower social harmony and low levels of collectivism are related to fewer prosocial behaviors and more problem externalization (38, 52, 57). Related longitudinal studies found that maternal social harmony negatively predicted 7th-grade adolescents' relational aggression in the 9th grade (38). As passive bystanding behaviors may encourage bullies and positively relate to delinquency (11), we assume that lower social harmony might be related to more passive bystanding behaviors during bullying.

In summary, higher parental support might facilitate more active defending behaviors through increased social harmony, whereas lower parental support might promote more passive bystanding behavior through reduced social harmony among Chinese adolescents. Previous research recommends integrating microsystem- and macrosystem-level factors from the perspective of the social ecological system. However, to our knowledge, no empirical study has explored the role of cultural values in the association between parental factors and bullying bystander behavior in non-Western cultural contexts.

### 1.3. The present study

**Figure 1:**
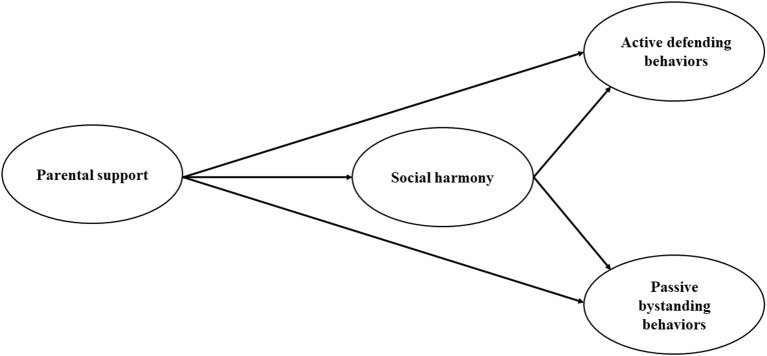
Hypothesized conceptual model.

Using a longitudinal design (Time 1 and Time 2; 17-month interval) and samples of Chinese adolescent student behavior, the overarching goal of this study was to examine the mediating role of social harmony on the association between parental support and active defending and passive bystanding behavior in bullying among Chinese adolescents. As Chinese culture highly values prosocial behaviors that may protect or promote social harmony, detrimental or bullying behaviors that use one's strength or dominant status to bully the weak may harm interpersonal harmony. Due to its specific cultural background, China may be an ideal place to explore these research questions. Thus, the following hypotheses are proposed (Figure 1):

**Hypothesis 1 (H1)**: Higher parental support is positively related to active defending behaviors among Chinese adolescents and negatively related to passive bystanding behaviors during bullying.
**Hypothesis 2 (H2)**: Social harmony mediates the positive relationship between parental support and active defending behaviors among adolescents.
**Hypothesis 2 (H3)**: Social harmony mediates the negative relationship between parental support and passive bystanding behaviors among adolescents.

## 2. Methods

### 2.1. Participants and procedures

Before commencing data collection, this study was approved by the Ethics Committee for Research at the first author's institution. The participants were adolescents in 7th grade from a junior high school in East China. Considering that boarding school students may differ from day school students in terms of the time and content of parent-child communication, only day school students were selected as participants in this study to reduce potential variability (58). All participants and their parents provided informed consent at the baseline assessment (Time 1), and all participants voluntarily participated in the study. A total of 812 responses were collected. However, data from four participants were excluded as they failed to complete the questionnaire (male = 431, 53.3%; mean age = 12.40, *SD* = 0.50). Before the start of Time 2, a new wave of COVID-19 hit the participants' city. Accordingly, all participants stayed at home as part of the local government's policy. The participants and teachers could communicate only through the mobile phones of the adolescents' parents. Therefore, requests for participation and consent during the new survey were not obtained in time. Among those who received a request for participation during the Time 2 survey, 514 gave their approval to be contacted for the second-wave study on time (Time 2) and fully completed the online questionnaires. Due to website malfunction or maloperation at the participants' end, 69 data points were deleted. Thus, the final sample encompassed 445 participants (male = 221, 47.4%; mean age = 14.41, *SD*= 0.51).

Following the time interval of previous related longitudinal studies (59, 60), data was planned to be measured during the 12 months students are in school. Data were collected online during November 2020 (T1) and April 2022 (T2). The actual time interval was 17 months, including 5 months of vacation (where schools were temporarily closed due to the COVID-19 prevention policy) and 12 months of school time. Teachers were instructed to use social networking software to explain the purpose of the investigation and the participants' rights. Both the parents and students were asked to provide written informed consent online. Once the parents' and students' consent was obtained, participants were instructed to access the web link of the relevant survey.

### 2.2. Measures

#### 2.2.1. Parental support

Parental support was evaluated using the parental social support scale, which was developed by Richards et al. (61), which comprises five items. Participants answered their perceived parental support behaviors on a five-point Likert 5-point scale (0 = strongly disagree to 4 = strongly agree; sample item: “My parents give me the right amount of affection”). Higher scores indicate a higher degree of parental support. Cronbach's alphas were 0.70 and 0.73 for T1 and T2 parental support, respectively.

#### 2.2.2. Social harmony

An eight-item social harmony adaptive version of the measurements was developed by Shuster et al., was used (38) (Sample item: “Harmonious interpersonal relationships in family and school”). Participants stated each item's importance in their lives on a five-point Likert scale (1 = not important at all; 5 = very important). Higher scores indicate a higher endorsement of social harmony values. Cronbach's alphas were 0.93 and 0.93 for T1 and T2 social harmony, respectively.

#### 2.2.3. Bullying bystander behaviors

Active defending behaviors and negative bystanding behaviors were evaluated using the six-item self-report Behaviors in Bullying Scale, which was developed by Pozzoli and Gini (11). Active (sample item: “I defend classmates who are hit or attacked.”) and passive bystander behaviors (sample item: “If I know that someone is excluded or isolated from the group, I act as if nothing has happened.”) were each evaluated using three items. Each item described one bystander behavior in one form of bullying (physical, verbal, and relational bullying). Participants stated how often (during the last 3 months) they had acted on the behavior described in each item on a four-point scale (1 = never, 4 = almost always). The Chinese version of this scale has been widely used in previous research and has shown good reliability and validity (47, 62, 63). For active defending behaviors, Cronbach's alpha was 0.77 for both T1 and T2. For passive bystanding behavior, Cronbach's alphas were 0.78 and 0.72 for T1 and T2, respectively.

### 2.3. Covariate variables

Participants were asked to report their gender (coded as 0 = female, 1 = male) as it may be associated with bullying bystander behaviors among adolescents (64).

### 2.4. Analysis plan

The Statistical Package for the Social Sciences 23.0 (SPSS, Inc., Chicago, IL, USA) and AMOS 23 software were used to analyze the data. First, Common method bias was tested and the Pearson's correlation analysis was used to calculate the association between the study variables at Times 1 and 2. Second, the construct reliability and coefficient of internal consistency. Third, confirmatory factor analysis (CFA) was performed to construct the measurement model. Fourth, structural equation modeling (SEM) was performed to examine the direct effects of parental support on active and passive bystander behaviors and the mediating effects of social harmony on the associations between parental support and the two forms of the bullying bystander behaviors of Chinese adolescents (i.e., active defending behaviors and passive bystanding behaviors). The normality test in this study (SKEW < 3) justified the use of maximum likelihood estimation in data analysis (65). We used the bias-corrected bootstrapping approach with a 95% confidence interval (66). We evaluated the goodness of fit of the measurement and structural models using the following indicators: chi-square/df, goodness-of-fit index (GFI), comparative fit index (CFI), standardized root mean square residual (SRMR), root mean square error of approximation (RMSEA) and the value of average variance extracted (AVE) (67). We included gender, T1 active defending behaviors, and T1 passive bystanding behaviors into the model as covariates. The pathway and model were reported using unstandardized path coefficients.

## 3. Results

First, the skewness and kurtosis of all the study variables were used to test the normal distribution of the data. All the variables fell within the acceptable range (skewness cutoff: 2.00; kurtosis cutoff: 7.00) (68). Second, Harman's single-factor test was applied to examine common method bias. The results showed that the factor loading of the first factor was 22.51%, indicating no significant common method bias in this study (69).

Third, the means, standard deviations, and correlation matrix of the variables were calculated, as shown in Table 1. There was a significant positive correlation between T1 parental support, T1 social harmony, and T1 active defense behaviors. T1 parental support was also significantly positively related to T2 parental support, T2 social harmony, and T2 active defending behaviors but negatively related to T2 passive bystanding behaviors (*ps* < 0.05), whereas the correlations between T1 parental support and T1 passive bystanding behaviors were non-significant. Additionally, T1 social harmony was significantly positively related to T1 and T2 active defending behaviors. However, no significant correlation was observed between T1 social harmony and T1 or T2 passive bystanding behaviors.

**Table 1:** Descriptive statistics and correlations (*N* = 445).

	**1**	**2**	**3**	**4**	**5**	**6**	**7**	**8**	**9**
1. T1 parental support	1								
2. T1 social harmony	0.23^**^	1							
3. T1 active defending behaviors	0.16^*^	0.22^*^	1						
4. T1 passive bystanding behaviors	−0.07	−0.09	−0.37^**^	1					
5. T2 parental support	0.44^**^	0.16^*^	0.07	−0.01	1				
6. T2 social harmony	0.19^**^	0.38^**^	0.15^**^	−0.05	0.24^**^	1			
7. T2 active defending behaviors	0.19^**^	0.17^**^	0.39^**^	0.19^**^	0.27^**^	0.27^**^	1		
8. T2 passive bystanding behaviors	−0.12^**^	−0.01	−0.21^**^	−28^**^	−0.16^**^	−0.14^**^	−0.47^**^	1	
9. Gender	−0.04	−0.03	−0.02	0.03	0.04	−0.07	−0.07	0.14^**^	1
*M*	3.94	4.33	2.92	1.30	3.90	4.13	2.67	1.98	1.53
SD	0.81	0.96	0.74	0.70	0.80	1.04	0.72	0.65	0.50

Gender is coded as 1 = Male, 2 = Female. The mean for child sex reflects the percentage of male students.

^*^p < 0.05.

^**^p < 0.01.

Fourth, composite reliability (CR) and Cronbach's α were applied to measure the reliability of the instrument, which showed good CR in this study (CR > 0.70) (70) (Table 2).

**Table 2:** Reliability results and AVE results of constructs.

	**CR**	**AVE**	**Cronbach's alpha**
1. T1 parental support	0.87	0.63	0.70
2. T1 social harmony	0.93	0.63	0.93
3. T1 active defending behaviors	0.80	0.58	0.77
4. T1 passive bystanding behaviors	0.85	0.65	0.78
5. T2 parental support	0.81	0.53	0.73
6. T2 social harmony	0.93	0.63	0.93
7. T2 active defending behaviors	0.76	0.52	0.77
8. T2 passive bystanding behaviors	0.79	0.55	0.72

CR, composite reliability; AVE, average variance extracted.

Fifth, CFA was performed to construct the measurement model. The model fit indices showed that the model fit the data well: χ^2^ = 477.74, *df* = 240, *p* < 0.001, χ^2^/*df* = 1.99, GFI = 0.92, IFI = 0.96, TLI = 0.95, CFI = 0.96, RMSEA = 0.05 [90% confidence interval (CI) = (0.04, 0.05)]. All standardized factor loadings were significant (*p* < 0.001). Moreover, none of the factor loadings were smaller than 0.4 (67). In addition, the value of average variance extracted (AVE) was calculated to check the validity of the measurement model (70), which indicated that the measurement model was effective (AVE >0.50) (Table 2). The measurement model was suitable for further analysis of the structural equation model.

**Figure 2:**
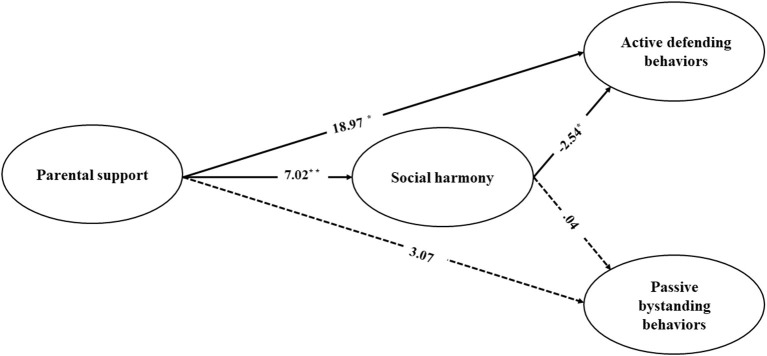
Mediation model of the association between T1 parental support and T2 behaviors of bullying bystanders (T2, active defending behaviors; T2, passive bystanding behaviors) *via* T2 social harmony with unstandardized beta weights and significance levels added. To simplify the presentation, the correlations between independent variables, and the correlation lines and predicting pathways involving covariates are not shown in the figure. Solid lines indicate relations that were significant at *p* < 0.05. Parameter estimates for pathways that were not statistically significant at *p* < 0.05 and *p* < 0.01 (two-tailed) are depicted as dashed lines in the figure. *p < 0.05, **p < 0.01.

Last, we established an indirect effects model to test our hypotheses. This model also fit the data well: χ^2^ = 449.51, *df* = 247, *p* < 0.001, χ^2^/*df* = 1.82, GFI = 0.92, IFI = 0.96, TLI = 0.96, CFI = 0.95, RMSEA = 0.04 [90% confidence interval (CI) = (0.04, 0.05)]. As Table 3 and Figure 2 showed that T1 parental support was a significant positive predictor of T2 active defending behaviors (β = 18.97, *p* < 0.05) but failed to relate to T2 passive bystanding behaviors (β = 3.07, *p* = 0.15). Moreover, T1 parental support was significantly and positively related to T2 active defending behaviors through the mediation of T2 social harmony [indirect effect = 17.85, 95% bias-corrected bootstrap confidence interval: (−53.03 to −7.26)]. T1 parental support was also significantly and positively related to T2 passive bystanding behaviors through the full mediation of T2 social harmony [indirect effect = −3.59, 95% bias-corrected bootstrap confidence interval: (−0.12.63 to −0.41)]. Figure 2 and Table 3 shows the path coefficients of the model.

**Table 3:** Unstandardized indirect effects from parental support to bystander behaviors (*N* = 445).

**Indirect paths**	**Unstandardized indirect effect**	**SE**	***p*-value**	**95% CI unstandardized indirect effect**
				**Boot LLCI Boot ULCL**
Parental support → social harmony → active defending behaviors	−17.85	13.23	0.001^**^	(−53.03, −7.26)
Parental support → social harmony → passive bystanding behaviors	−3.59	3.31	0.000^**^	(−12.63, −0.41)

CI, confidence interval; LLCI, low limit; ULCL, upper limit.

These values are based on unstandardized path coefficients. All parameter estimates and significance tests are based on 5,000 bootstrapped samples.

Significant effects are determined by both 95% CI that does not include zero and *p* < 0.05.

^*^p < 0.05.

^**^p < 0.01.

## 4. Discussion

We constructed a mediation model to explore how parental support is associated with bullying bystanding behavior among Chinese adolescents. The results showed that parental support was positively linked to adolescents' active defending behaviors *via* the partially mediating effect of social harmony, but negatively related to passive bystanding behaviors *via* the fully mediating effect of social harmony.

### 4.1. Direct effect of parental support on bullying bystander behaviors

There was a clear direct relationship between parental support and Chinese adolescents' active defending behaviors, thus supporting **H1**. These results are consistent with the extant literature, which suggests that parental warmth and support may facilitate more secure attachments and is related to more prosocial behaviors (44, 45). Parents' warmth and support may encourage children to develop more positive problem-solving skills (71), which helps them adopt more prosocial and effective behaviors to solve bullying incidents (25). This finding is consistent with that of a previous study (54). Our findings highlight the important role of parents in adolescents' socialization and social behavior development (40). Our findings also support and expand attachment theory, which suggests that parental warmth and support could promote the development of a more prosocial internal working model and social behaviors for children (43). However, the direct links between parental support and Chinese adolescents' passive bystanding behaviors in bullying were not significant, which does not support **H1**. These results are consistent with those of previous cross-sectional studies, which found that parental security attachment could fail to extend to outsider behaviors in bullying but is positively related to active defending behaviors (18). One explanation is that maternal and paternal social support may have different effects on passive bystanding behaviors. A previous study found that only attachment to the mother (not father) was significantly related to predictors of passive bystanding behaviors (72). Future studies should examine these explanations by adopting a longitudinal design and testing the potential unique effects of maternal and paternal social support on bullying bystander behaviors.

### 4.2. Mediating role of social harmony

Chinese culture highly values social harmony and prosocial behavior. Although many studies have discussed parental practice and aggression or bullying behaviors in Chinese culture (73, 74), this is the first empirical longitudinal study to investigate bullying bystander behaviors in the Chinese context, showing the mediating role of cultural factors. The mediating effects of social harmony on the associations between parental support and active defending or passive bystanding behaviors were significant, thus supporting **H2**. These findings support and extend the social-ecological system theory by highlighting the important roles of microsystem and macrosystem (social harmony) factors in Chinese adolescents' bullying bystander behaviors. Moreover, the findings support contextual development models by illustrating that parental warmth and support can promote culturally desirable behaviors through the development of social harmony values (38, 75, 76). Consequently, adolescents who internalized social harmony exhibited more active defending behaviors. Surprisingly, we failed to find any mediation effects for social harmony on passive bystanding behaviors. Besides the potentially different social support roles of mothers and fathers', one reason may be that there are differing intentions and views about the function of outsider behaviors in bullying. Some Chinese adolescents may perceive that if they stop bullying, the peer groups will experience more conflict and further harm their social harmony. Therefore, they prefer not to intervene. In other words, future studies could explore Chinese adolescents' views on passive bystanding behaviors in the promotion of social harmony.

The pathway results in the present study's mediation model should be noted. We found that social harmony significantly predicted Chinese adolescents' active defending behaviors, which supports and expands Schwartz's values theory (77). As mentioned, Schwartz's values theory (basic values) does not mention social harmony (77, 78), and the mainstream scale to measure related collectivistic values does not include the component of social harmony either (53, 55, 70). Our findings could facilitate fruitful research on cultural values and provide evidence for the development of new cultural value theories.

Most bullying bystander research examines Western and individualistic cultures, hindering the understanding of bullying bystanders in more diverse contexts. In traditional Chinese culture, children are trained to depend on and obey their parents. Thus, parental social support may affect Chinese adolescents' social development more stronger. In addition, Chinese culture highlights the importance of interpersonal harmony and scorns behaviors that harm it. Thus, a higher social harmony value may induce stronger active defending behaviors in Chinese adolescents (79, 80).

Taken together, this research could help us understand the role of cultural factors among bullying bystanders, which is beneficial for building a more comprehensive, culturally diverse theoretical model.

### 4.3. Strengths, limitations, and future directions

Our study has two main theoretical implications. First, it elucidates how parental support is associated with bullying bystander behaviors among adolescents from collectivistic cultures. Moreover, it extends existing knowledge by uncovering the merging effects of different ecological system-level factors (81). Specifically, we highlight the important role of a macrosystem factor (social harmony).

Second, as most bullying bystander theories focus on situational or social-cognitive process factors in bullying bystanders (24, 82–86), our findings highlight the necessity to add cultural-specific factors to these theories. Future studies could use our evidence to build more comprehensive and culturally sensitive bullying bystander theories.

Our findings also provide two practical implications. First, we underscore the necessity of adding a parental practice training curriculum in bystander programs. This could help schools build evidence-based bullying prevention programs by adding parental training components to boost parental warmth and support. Second, the mediating effect of cultural value (social harmony) implies that bullying bystander intervention programs could involve sessions about Chinese adolescents' increased endorsement of social harmony and further encouraging active defending behaviors. However, this study has a few limitations. First, we only examined defending and outsider bystanding behaviors. It is unclear whether the other types of bystander behaviors would also be affected by parental support (e.g., indirect defending, reinforcing). Future studies should compare the effects of parental support on multiple forms of bystander behaviors (87). Second, this study focuses on the bystander behaviors of bullying in school but did not include cyberbullying bystander behaviors. Future studies should examine these findings in cyberbullying contexts. Third, due to the COVID-19 pandemic, we were unable to contact all participants in time for the Time 2 survey, which led to sample loss. Future longitudinal studies should be conducted in areas with no confirmed COVID-19 cases to reduce sample loss. Fourth, all data in this study were obtained through self-reports from day school students. Moreover, this study did not compare the effects of paternal and maternal support. Future studies could include father, mother, and student reports through more representative samples and test gender-sensitive issues. Future studies may also extend the literature by comparing potential differences between boarding school and day school students (58). While this study employed a quantitative method, future studies could adopt a mixed-methods approach (quantitative and qualitative), which may deepen our understanding of bullying bystander behaviors (88).

## 5. Conclusions

Bullying and bullying victimization are serious social problems affecting children and adolescents' health and contributing to the governmental public health financial burden worldwide. Bystander behavior significantly contributes to the bullying process. However, it is unclear how family factors are associated with adolescents' active defense and passive bystanding behaviors in non-Western cultural contexts. This longitudinal study adds empirical evidence on how cultural value (social harmony) mediates the associations between parental support and multiple bullying bystanders based on the social-ecological system theory framework. This finding highlights the importance of fully considering cultural-related factors in the theoretical development of bullying and intervention program design.

## Data availability statement

The original contributions presented in the study are included in the article/supplementary material, further inquiries can be directed to the corresponding author.

## Ethics statement

The studies involving human participants were reviewed and approved by the Institutional Review Board (or Ethics Committee) of the School of Smart Education, Jiangsu Normal University. Written informed consent to participate in this study was provided by the participants' legal guardian/next of kin. Written informed consent was obtained from the individual(s), and minor(s)' legal guardian/next of kin, for the publication of any potentially identifiable images or data included in this article.

## Author contributions

Conceptualization: YZ and XJ. Methodology and funding acquisition: XJ and JW. Validation: JW and YZ. Formal analysis, investigation, resources, visualization, and project administration: YZ. Writing—original draft preparation and writing—review and editing: YZ, XJ, and JW. Supervision: XJ. All authors have read and agreed to the published version of the manuscript.
